# Racial Differences in the Prognosis and Survival of Cutaneous Melanoma From 1990 to 2020 in North America: A Systematic Review and Meta-Analysis

**DOI:** 10.1177/12034754211052866

**Published:** 2021-10-22

**Authors:** Megan Lam, Jie Wei Zhu, Angie Hu, Jennifer Beecker

**Affiliations:** 1 12362 Faculty of Medicine, Michael G. DeGroote School of Medicine, Hamilton, ON, Canada; 2 2129 Faculty of Science, University of Calgary, AB, Canada; 3 Division of Dermatology, University of Ottawa, ON, Canada; 4 153195 The Ottawa Hospital, ON, Canada; 5 Ottawa Research Institute, ON, Canada; 6 Probity Medical Research Inc., Waterloo, ON, Canada

**Keywords:** melanoma, socioeconomic, race, survival, prognosis

## Abstract

**Background:**

Factors influencing the difference in the diagnosis and treatment of melanoma in racial minority groups are well-described in the literature and include atypical presentations and socioeconomic factors that impede access to care.

**Objective:**

To characterize the differences in melanoma survival outcomes between non-Hispanic white patients and ethnic minority patients in North America.

**Methods:**

We conducted searches of Embase via Ovid and MEDLINE via Ovid of studies published from 1989 to August 5, 2020. We included observational studies in North America which reported crude or effect estimate data on patient survival with cutaneous melanoma stratified by race.

**Results:**

Forty-four studies met our inclusion criteria and were included in this systematic review. Pooled analysis revealed that black patients were at a significantly increased risk for overall mortality (HR 1.42, 95% CI, 1.25-1.60), as well as for melanoma-specific mortality (HR 1.27, 95% CI, 1.03-1.56). Pooled analyses using a representative study for each database yielded similar trends. Other ethnic minorities were also more likely report lower melanoma-specific survival compared to non-Hispanic white patients.

**Conclusion:**

Our results support findings that melanoma patients of ethnic minorities, particularly black patients, experience worse health outcomes with regards to mortality. Overall survival and melanoma-specific survival are significantly decreased in black patients compared to non-Hispanic white patients. With the advent of more effective, contemporary treatments such as immunotherapy, our review identifies a gap in the literature investigating present-day or prospective data on melanoma outcomes, in order to characterize how current racial differences compare to findings from previous decades.

## Introduction

While the incidence of cutaneous melanoma in minority populations are reported to be lower than in non-Hispanic white populations, age-adjusted incidence rates of cutaneous melanoma of all stages are increasing.^1,2^ Melanoma accounts for approximately 75% of skin cancer deaths, and early detection plays an important role in reducing mortality risk.^3^

Several studies have identified disparities in the diagnosis and treatment of melanoma in racial minority patients compared to non-Hispanic white patients,^4-6^ and the presentation of cutaneous melanoma in minority groups is more frequently characterized as atypical in general appearance, location, and histologic subtype.^6^ Socioeconomic disparities that disproportionately affect black populations, including residing in lower socioeconomic status neighborhoods and being uninsured, are associated with later stage at cancer diagnosis and a greater risk of death.^7-10^ We conducted this systematic review and meta-analysis to assess and characterize the differences in melanoma survival outcomes between non-Hispanic white and black patients, as well as other ethnic minority groups.

## Material and Methods

This systematic review was performed in accordance with the Preferred Reporting Items for Systematic Reviews and Meta-Analyses (PRISMA) guidelines. The protocol of this review was prospectively registered in the Prospective Register of Systematic Reviews (PROSPERO; CRD42020202891).

### Search Strategy

We conducted a comprehensive search of Embase via Ovid and MEDLINE via Ovid on August 5, 2020. Our search strategy is comprised of key terms for race and socioeconomic factors, melanoma, and observational study designs, with guidance from an experienced librarian (Mendeley Supplemental Tables S1-S2). Our search was limited to human studies and English records, as well as studies published from 1989 to 2020, inclusive. Additionally, we manually searched citations of included studies and relevant review articles for additional studies not included in our original search.

### Study Selection and Data Abstraction

We included observational studies (cohort, cross-sectional, or case-control studies) of melanoma patients which reported either (i) crude survival or mortality prevalence rates out of total patients with cutaneous melanoma, or (ii) effect estimate data on survival or mortality of cutaneous melanoma, stratified by more than one race or ethnicity. Studies conducted outside of North America were excluded, as stated previously.^11,12^ Additionally, we excluded studies that reported (i) only one race, (ii) insufficient data to determine the effect estimate, relative survival, or denominator for prevalence,^13^ or (iii) outcomes not specific to cutaneous melanoma patients.

All retrieved titles and abstracts were screened for relevance and full texts assessed for eligibility independently and in duplicate (pairs of M.L., J.Z., and A.H.). Discrepancies between reviewers were discussed until consensus was reached. The following data were extracted in duplicate (M.L. and J.Z.) from individual studies using a standardized form: study characteristics (author, year of publication, disease, funding, patient source, inclusion criteria), patients demographics (number of patients, race groups, age range), melanoma characteristics (tumor stage, Breslow thickness, local or distant metastasis, melanoma type), and outcomes (type of survival/mortality, no. of deaths, % survival, effect estimate, adjusted variables).

### Quality Assessment of Studies

Risk of bias for included studies was assessed using the NIH National Heart Lung and Blood Institute Quality Assessment Tool for cohort and cross-sectional studies, and the Quality Assessment Tool for case-control studies was planned to be used but was not needed as there weren’t any case control studies included. A quality rating of good, fair, or poor was assigned independently by 2 reviewers (M.L. and J.Z.) based on a set of 14 criteria, and discrepancies were discussed until full consensus was reached.

### Statistical Analysis

The proportions of studies reporting lower overall survival and melanoma-specific survival in ethnic minority patients were calculated. Random-effects inverse variance meta-analyses were performed where possible to generate pooled hazard ratios for risk of mortality for patients of ethnic minorities compared to non-Hispanic white patients. In the case where multiple studies drew from the same database of patients, only representative studies which drew patients from the longest length of time from their respective databases. Additionally, sub-analyses eliminating all studies using data from any national cancer database were performed to further reduce risk of double-counting patient data. Estimates reported from the most adjusted model were used when available. Statistical heterogeneity between studies were assessed using the I^2^ statistic. All analyses were conducted using Review Manager version 5.3 (Cochrane Collaboration, Software Update, Oxford, United Kingdom). *P* values of less than 0.05 were considered statistically significant.

## Results

Our initial search yielded a total of 4906 records. Upon removal of 1220 duplicate records, 3686 records were screened based on titles and abstracts. From these, 368 records were assessed for full-text eligibility. We excluded 324 full-text records, including 102 records excluded due to irrelevant study design, 27 records for not examining outcomes specific to cutaneous melanoma, 70 records for not stratifying by race or only examining one race, and 77 records that did not examine outcomes of survival or mortality. Ultimately, 44 studies met our inclusion criteria and were included in this systematic review (Supplemental Figure S1).^2,4,6,10,14-53^

Most studies were published from 2011 and onwards (*n* = 35), reported a public source of funding (*n* = 19), and used patient data drawn from national cancer databases (*n* = 35). Eleven studies included adult patients exclusively and 2 studies included only pediatric patients. Most studies (26 studies) were assigned a quality assessment rating of “fair” and 11 studies were assigned a rating of “good”. The 7 studies assigned a “poor” rating were predominantly found to have insufficiently defined study populations, outcome measures, and confounding variables (Table 1).

**Table 1:** Overview of Study and Patient Characteristics for Studies Included in the Systematic Review.

	No. of studies (%)^a^
***Study characteristics***	
Year published	
1990–1994	1 (2)
1994–1999	1 (2)
2000–2004	1 (2)
2005–2009	5 (11)
2010–2014	10 (23)
2015–2020	26 (59)
Funding	
Public	19 (43)
Public and private	2 (5)
None	6 (14)
Not reported	17 (39)
Patient source	
National database	35 (80)
SEER	31 (70)
NCDB	4 (9)
State registry data	4 (9)
Hospital data	2 (5)
Other database	3 (7)
NIH Quality Assessment	
Good	12 (27)
Fair	25 (57)
Poor	7 (16)
***Patient characteristics***	
Minority groups	
Black	37 (84)
Asian	22 (50)
Pacific islander	15 (34)
NaBlacktive	13 (30)
Hispanic	23 (52)
Age range	
Adult only	11 (25)
Peds	2 (5)
Both	5 (11)
NR	26 (59)

^a^Out of the 44 included studies.

Of the 44 studies included, there were a total of 3,168,108 patients. Thirty-seven studies included black patients (*n* = 23578). With respect to study outcomes, 28 studies reported effect estimates for survival stratified by any race. 29 studies reported disease-specific survival/mortality, 20 studies reported overall survival/mortality, and 3 studies reported relative survival data.

### Outcomes for Black Patients Compared to Non-Hispanic White Patients

In total, 33 (75%) studies compared non-Hispanic white and black patient survival, with 27 of these studies reporting worse outcomes for black patients. Five studies reported AJCC cancer stages for black patients, with weighted mean % of patients at stage I (*n* = 501 patients, 52.6%) , stage II (*n* = 223, 24.1%), stage III (*n* = 123, 16.3%), and stage IV (*n* = 101, 13.2%).^4,6,15,19,33^ Seven studies comparing non-Hispanic white and black survival reported histological subtype stratified by race, with total patients and mean proportion of patients as follows: nodular melanoma (black: *n* = 1090, 10.0%; non-Hispanic white: *n* = 72029, 9.1%), lentigo maligna melanoma (*n* = 196, 3.7%; *n* = 75631, 7.6%), superficial spreading melanoma (*n* = 1611, 19.0%; *n* = 312754, 39.3%), and acral lentiginous melanoma (*n* = 2107, 19.4%; *n* = 8141, 0.99%).^2,4,6,18,23,34,45^ The most common subtype reported in black patients were superficial spreading melanoma in 4 studies, and acral lentiginous melanoma in 3 studies, while superficial spreading melanoma was the most commonly reported subtype for non-Hispanic white patients among all 7 studies. Malignant melanoma with subtype not otherwise specified was reported in a greater proportion of black patients (*n* = 2825, 53.9%) than white patients (*n* = 483,065, 48.0%). Six studies reported the anatomic site of the primary melanoma, all of which reported the lower extremities as being the most common site for black patients and the trunk as the most common site for non-Hispanic white patients.^2,4,6,34,43,45^

Of the 21 studies that reported effect estimates for mortality risk for black patients with non-Hispanic white patients as the reference group, 14 adjusted for cancer stage, 8 for histologic subtype, and 7 for anatomic site. Four studies adjusted for tumor thickness, while 3 studies adjusted for ulceration status. Socioeconomic status was included in adjustments in 7 studies, of which 4 reported adjusting for income specifically and 2 reported adjusting for education level (Table 2).

**Table 2:** Adjusted Variables in Studies Reporting Effect Estimates for Mortality Risk for Black Patients for Studies Included in the Meta-Analysis.

Variables adjusted	No. of studies (%)^a^
Sex	13 (62)
Age	11 (52)
Marital status	5 (24)
Age at diagnosis	2 (10)
Insurance	1 (5)
Socioeconomic status	7 (33)
*Income*	*4* (*19*)
*Education*	*2* (*10*)
Cancer stage	14 (67)
Histologic subtype	8 (38)
Anatomic site	7 (33)
Tumour thickness	4 (19)
Tumour ulceration	3 (14)
Lymph node involvement	3 (14)
Treatment type	5 (24)

^a^Out of 21 studies reporting effect estimates for mortality risk for black vs non-Hispanic white patients.

### Overall Survival for Black Patients Compared to Non-Hispanic White Patients

All 5 studies measuring overall survival percentages reported decreased survival for black patients compared to non-Hispanic white patients.^2,18,23,34,40^ However, one study reported significantly increased survival for black patients following adjustment for income (Supplemental Table S3).^23^

**Figure 1:**
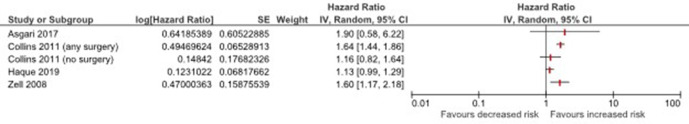
Risk of overall mortality in black patients compared to non-Hispanic white patients in representative studies.

Twelve studies reported effect estimates and confidence intervals for risk of mortality from any cause for black patients compared to non-Hispanic white patients. All studies reported an increased risk of death for black patients. Pooled analysis of effect estimates of all studies resulted in a significantly increased risk of mortality from any cause for black patients, with a pooled HR of 1.42 (95% CI, 1.25-1.60). As multiple studies drew patient data from the same database, we pooled effect estimates from a representative study from each database with the longest timeframe from which patient data were drawn (HR 1.38, 95% CI, 1.10-1.73) (Figure 1).^2,6,28,52^ We also performed sensitivity analyses removing any studies using patient data from the Surveillance, Epidemiology, and End Results (SEER) program database (HR 1.29, 95% CI, 1.07-1.56),^6,28,51,52^ and removing any studies with patient data from any national cancer database (HR 1.62, 95% CI, 1.20-2.19).^6,52^ Overall, results of our meta-analyses suggest that black melanoma patients are significantly more likely to experience death from any cause compared to non-Hispanic white patients.

### Melanoma-Specific Survival for Black Patients Compared to Non-Hispanic White Patients

Twelve studies reported melanoma-specific survival percentages,^2,15,20,29-32,34,36,45,50,53^ and among the 11 studies reporting 5-year survival, 9 studies reported decreased survival for black patients. Of the 3 studies reporting 10-year survival, 1 study reported decreased survival for black patients (Supplemental Table S3).

Thirteen studies reported effect estimates for risk of melanoma-specific death for black patients compared to non-Hispanic white patients, of which 4 studies reported a decreased risk of melanoma-specific mortality for black patients.^2,31,41,43^ Collins et al. reported that while black patients experienced a significantly increased risk of melanoma-specific death upon receiving surgical treatment, black patients also experienced a decreased risk of death in the nonsurgical treatment group, although this association was not statistically significant.^2^ Similarly, Jemal et al. reported a nonsignificant decreased risk of melanoma-specific death for black male patients, but a significantly increased risk of melanoma-specific death for black female patients, compared to female non-Hispanic white patients.^31^ Ward-Peterson reported a significantly decreased melanoma-specific mortality risk for black patients compared to non-Hispanic white patients (HR 0.7, 95% CI, 0.6-0.8, *P* < .001) in the most adjusted model that accounted for both site and stage at diagnosis.^43^

**Figure 2:**
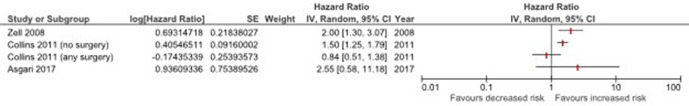
Risk of melanoma-specific mortality in black patients compared to non-Hispanic white patients in representative studies

Pooled analysis of effect estimates from all studies revealed a significantly increased risk of melanoma-specific mortality for black patients, with a pooled HR of 1.27 (95% CI, 1.03-1.56). Using a representative study from each database also found a stronger association (HR 1.45, 95% CI, 1.01-2.07) (Figure 2).^2,6,52^ Following removal of studies with data from any national cancer database, we observed a stronger association between melanoma-specific mortality and black patients (HR 2.04, 95% CI, 1.35-3.07).,^6,52^Our results support an overall increased melanoma-specific mortality in black patients compared to non-Hispanic white patients.

### Melanoma-Specific Survival for Other Ethnic Minorities Compared to Non-Hispanic White Patients

Twenty-three studies reported 62505 Hispanic patients,^4,6,10,15,19-22,24,25,27,30,31,36-38,43-45,50-53^ with 10 studies reporting melanoma-specific survival,^4,15,19,20,30,31,36,45,50,53^ where all but 2 studies^50,53^ reported lower survival for Hispanic patients compared to non-Hispanic white patients.

Twenty-one studies included Asian patients,^4,6,10,15,19-22,30,31,36-39,41,42,44,45,50,52,53^ with 17 of these studies combining patients of Asian and Pacific Islander race into 1 subgroup, to a total of 18878 Asian and Pacific Islander patients. Ten studies reported melanoma-specific survival,^4,15,20,30,31,36,39,45,50,53^ with all but 2 studies^50,53^ reporting lower survival for Asian and Pacific Islander patients compared to non-Hispanic white patients (Supplemental Table S4). The 2 studies that reported a higher survival had in Asian and Pacific Islander patients also reported higher survival for Hispanic patients, and included only a small number of patients in each minority group (*n* = 19 Asian/Pacific Islander patients, *n* = 97 Hispanic patients).^50,53^

Thirteen studies included American Indian patients,^4,20,21,30,31,36-38,41,42,44,45,53^ with 9 studies also including Alaskan Native patients in this group, to a total of 2239 patients. Seven studies reported melanoma-specific survival for American Indian and Alaskan Native patients,^4,20,30,31,36,45,53^ with only 1 study reporting higher survival in American Indian or Alaskan Native patients compared to non-Hispanic patients.^53^

There was insufficient data to pool effect estimates using a representative study from each database for these ethnic minority groups.

## Discussion

A total of 44 studies were included, of which 33 studies compared survival or mortality outcomes for black patients with those of non-Hispanic white melanoma patients. Pooled analyses indicate a statistically significant difference, with decreased overall survival and melanoma-specific survival for black patients. Melanoma-specific survival for other ethnic minorities was also more frequently reported as lower compared to non-Hispanic white patients. A gap in the present literature exists where more recent studies omit an updated examination of current disparities compared to findings from previous decades.

Reduced overall survival for ethnic minority patients compared to non-Hispanic white patients may be attributed to several contributing factors. First, a longer time to diagnosis and subsequently a diagnosis at more advanced stages can lead to a poorer prognosis.^19,54,55^ A study of over 100,000 patients by Dick et al. found black patients were significantly more likely to present with advanced-stage disease, even after adjusting for tumor characteristics and patient demographic factors.^56^ A recent retrospective review from the National Cancer Database also showed that black patients had longer time from diagnosis to definitive surgery, which also might influence survival.^57^ Second, socioeconomic status differences may lead to increased barriers that limit access to medical care in minority populations. Communities of lower socioeconomic status also tend to have a decreased density of dermatologists, further reducing access to care.^58^Third, acral lentiginous melanoma comprises a higher proportion of total melanoma in patients of black, Hispanic, and Asian race. This subtype is associated with increased tumor thickness and more advanced stage at presentation,^15,59,60^ and in our analyses we found that black patients were more likely to present with acral lentiginous melanoma (19.4%) compared to white patients (0.99%). Black patients were also more likely to be reported as having a malignant melanoma of unspecified subtype compared to non-Hispanic white patients. Genetic differences may cause melanoma tumors to present more aggressively or nontraditionally in certain populations.

Unfortunately, many of the studies published more recently however continued to use retrospective patient data prior to 2011, which obscures trends in how racial disparities have changed over time. With the advent of more effective, contemporary treatments such as immunotherapy, it will be important to examine how racial differences in outcomes in North America are affected. The study by Haque et al. highlights that while immunotherapy was associated with improved overall survival, African American patients were less likely to receive immunotherapy.^28^ Interestingly, the study by Al-Qurayshi et al. in 2018 found no statistically significant racial differences in immunotherapy usage, but rather, that those without private insurance translated into worse outcomes in mortality, and seemed to be a more significant contributing factor.^51^ Recent decades have also seem the implementation of various measures to improve health outcomes and access to healthcare for marginalized groups including increased education around the presentation of common skin conditions in ethnic skin types and a greater inclusion of articles on skin of color.^61-63^ However, overall inclusion is still low and darker skin types are still underrepresented in mainstream dermatology textbooks and educational materials.^64,65^ Ultimately, health outcomes for melanoma patients of racial backgrounds need to be revisited with a modern lens in order to better characterize current racial differences in outcomes.

### Limitations

Our study had several limitations. Our review was limited to studies conducted in North America, with the large majority of studies from the United States, and while this reduced heterogeneity of sociocultural factors, studies from other multi-cultural countries outside of North America offer important insight that may be applicable to the general population. Additionally, although we performed sensitivity analyses to remove studies using data from national cancer databases, pooled effect estimates may be exaggerated as the majority of studies that were included in this review used patient data from the SEER database. It must also be mentioned that the definitions of black and non-Hispanic white are open to subjectivity, and may differ between or even within studies. Lastly, most of the included studies were based on patient data obtained from national-level cancer databases. Sixteen percent of included studies were assigned a rating of “poor” during quality assessment, largely due to the limited nature of retrospective database analysis, and limited population description and justification.

### Conclusion

Overall, our results suggest that differences in health outcomes for ethnic minority melanoma patients, particularly black patients, continue to exist. While studies included in this review reported mortality among patients diagnosed with melanoma, additional large-scale epidemiological studies to better estimate case fatality rate will be valuable. Our review identified a paucity of literature investigating present-day or prospective data on melanoma outcomes, and as patients receive more effective, contemporary treatments, it will be important to continue to examine how healthcare professionals can seek to improve outcomes and reduce barriers to care for marginalized populations.

## Supplemental Material

Supplementary Material 1 - Supplemental material for Racial Differences in the Prognosis and Survival of Cutaneous Melanoma From 1990 to 2020 in North America: A Systematic Review and Meta-Analysis Click here for additional data file. Supplemental material, Supplementary Material 1, for Racial Differences in the Prognosis and Survival of Cutaneous Melanoma From 1990 to 2020 in North America: A Systematic Review and Meta-Analysis by Megan Lam, Jie Wei Zhu, Angie Hu and Jennifer Beecker in Journal of Cutaneous Medicine and Surgery
