# Supplementary material for: Racial Differences in the Prognosis and Survival of Cutaneous Melanoma From 1990 to 2020 in North America: A Systematic Review and Meta-Analysis
Source: J Cutan Med Surg. 2021 Oct 22;26(2):181–8. doi: 10.1177/12034754211052866 (PMC8950707; doi:10.1177/12034754211052866)
Supplement: Supplementary Material 1 - Supplemental material for Racial Differences in the Prognosis and Survival of Cutaneous Melanoma From 1990 to 2020 in North America: A Systematic Review and Meta-Analysis [file sj-docx-1-cms-10.1177_12034754211052866.docx]

**SUPPLEMENTAL MATERIAL**

**Racial differences in the prognosis and survival of cutaneous melanoma from 1990 to 2020 in North America: a systematic review and meta-analysis**

Megan Lam, BSc^1^, Jie Wei Zhu, BHSc^1^, Angie Hu, BSc^2^, Jennifer Beecker, MD, CCFP(EM), FRCPC^3,4,5,6†^

^1^ Michael G. DeGroote School of Medicine, Faculty of Medicine, Hamilton, Ontario, Canada

^2^ Faculty of Science, University of Calgary, Calgary, Alberta, Canada

^3^ University of Ottawa, Division of Dermatology, Ottawa, ON, Canada

^4^The Ottawa Hospital, Ottawa, ON, Canada

^5^Ottawa Research Institute, Ottawa, ON, Canada

^6^Probity Medical Research Inc., Waterloo, ON, Canada

**Table S1:** Search strategy for MEDLINE via Ovid

| # | searches |
| --- | --- |
| 1 | Socioeconomic Factors/ |
| 2 | socioeconom*.mp. |
| 3 | race*.mp. |
| 4 | Continental Population Groups/ |
| 5 | Race Factors/ |
| 6 | racial*.mp. |
| 7 | Minority Groups/ |
| 8 | Minority Health/ |
| 9 | ethnic*.mp. |
| 10 | African Americans/ |
| 11 | Asian*.mp. |
| 12 | Caucasian*.mp. |
| 13 | Hispanic Americans/ |
| 14 | Indigenous Peoples/ |
| 15 | OR/ {1-14} |
| 16 | melanoma/ |
| 17 | melano*.mp. |
| 18 | 16 OR 17 |
| 19 | Observational study/ |
| 20 | observation*.mp. |
| 21 | population*.mp. |
| 22 | nation*.mp. |
| 23 | Case-Control Studies/ |
| 24 | Cohort studies/ |
| 25 | Case-control.mp. |
| 26 | case?control.mp. |
| 27 | Cohort*.mp. |
| 28 | Observational study.pt. |
| 29 | Cross-Sectional Studies/ |
| 30 | OR/ {19-29} |
| 31 | 15 AND 18 AND 30 |
| 32 | 31 (limit to English and human studies) |
| 33 | 32 (limit to yr=“1989-2021”) |

Note: / - MeSH search, * - truncation, exp – explode

**Table S2:** Search strategy for Embase via Ovid

| # | searches |
| --- | --- |
| 1 | social status/ |
| 2 | socioeconom*.mp. |
| 3 | race/ |
| 4 | race difference/ |
| 5 | race*.mp. |
| 6 | racial*.mp. |
| 7 | minority group/ |
| 8 | minority health/ |
| 9 | minorit*.mp. |
| 10 | ethnic difference/ |
| 11 | ethnic*.mp. |
| 12 | “social determinants of health”/ |
| 13 | African American/ |
| 14 | Asian.mp. |
| 15 | Caucasian.mp. |
| 16 | Hispanic.mp. |
| 17 | Indigenous people/ |
| 18 | OR/ {1-17} |
| 19 | exp melanoma/ |
| 20 | melano*.mp. |
| 21 | 19 OR 20 |
| 22 | Observational study/ |
| 23 | observation*.mp. |
| 24 | population*.mp. |
| 25 | nation*.mp. |
| 26 | Case control study/ |
| 27 | Cohort analysis/ |
| 28 | case control.mp. |
| 29 | case-control.mp. |
| 30 | cohort*.mp. |
| 31 | cross-sectional/ |
| 32 | OR/ {22-31} |
| 33 | 18 AND 21 AND 32 |
| 34 | 33 (limit to English and human studies) |
| 35 | 34 (limit to yr=”1989-2021”) |

Note: / - MeSH search, * - truncation, exp – explode

**Figure S1**: Search methodology flow diagram

EMBASE **(n=3362)**

MEDLINE **(n=1544)**

Studies identified through database searching **(n=4906)**

Duplicate records removed

**(n=1220)**

Records screened based on titles and abstracts

**(n=3686)**

Records excluded **(n=3318)**

Full-text records assessed for eligibility **(n=368)**

Full texts excluded **(n=324)**:

Conference abstract or poster (n=93)

Case report or case series (n=1)

Letter/editorial/commentary without original data (n=3)

Review article (n=5)

Not specific to cutaneous melanoma (n=27)

Not stratified by race (n=70)

Irrelevant outcomes (n=77)

Not in North America (n=25)

Insufficient statistical data (n=16)

Duplicate (n=5)

No English translation (n=1)

No full text (n=1)

Total records included based on full-text eligibility

**(n=44)**

**Table S3:** Study and patient characteristics for studies examining overall survival between black and white patients for studies included in the meta-analysis

| *Study* | *Patient data source* | *Patient age range* | *Black n(%)* | *White n(%)* | *Survival length* | *Black survival %* | *White survival %* | *NIH Quality Assessment* |
| --- | --- | --- | --- | --- | --- | --- | --- | --- |
| *Overall survival* | | | | | | | | |
| Al-Qurayshi, 2018 | NCDB, 2004-2012 | Adult | 74 (1.2) | 6005 (97.4) | NA | NR | NR | Fair |
| Asgari, 2017 | KPNC, 2004-2012 | NR | 5 (4.1) | 92 (74.8) | NA | NR | NR | Good |
| Berkman, 2017 | SEER, 1973-2011 | Children and young adults | 76 (0.4) | 16326 (94.9) | NA | NR | NR | Fair |
| Collins, 2011 | SEER, 1973-2004 | NR | 789 (0.5) | 148833 (98.5) | 5-year | 46.3 | 55.8 | Good |
|  |  |  |  |  | 10-year | 41.2 | 49.8 |  |
| Culp, 2019 | SEER/NPCR, 2011-2015 | NR | 2848 | NR | 5-year | 85.5 (localized), 52.8 (regional), 19.0 (distant) | 97.5 (localized), 63.8 (regional), 19.8 (distant) | Fair |
| Dawes, 2016 | SEER, 1992-2009 | NR | 509 (0.5) | 91572 (94.8) | NA | NR | NR | Fair |
| Du, 2011 | SEER, 1973-2003 | ≥25 | 922 (7.0) | 10749 (81.2) | NA | NR | NR | Good |
| Ezaldein, 2015 | SEER | NR | 1611 (0.5) | 289554 (99.4) | 5-year | 55.17 | 55.17 | Poor |
| Haque, 2019 | NCDB, 2004-2014 | ≥18 | 271 (1.7) | 15340 (96.2) | NA | NR | NR | Fair |
| Mahendraraj, 2017 | SEER, 1988-2011 | NR | 1106 (0.5) | 212721 (99.5) | 5-year | 70.0 | 89.0 | Poor |
| Singh, 2016 | SEER, 1973-2012 | NR | 259 (0.3) | 92750 (96.9) | 5-year | 94.6 | 95.1 | Fair |
| Trofymenko, 2018 | SEER, 2003-2012 | NR | 90 (0.2) | 41284 (95.0) | NA | NR | NR | Fair |
| Ward-Peterson, 2016 | SEER, 1982-2011 | ≥18 | 3070 (1.7) | 169844 (94.4) | NA | NR | NR | Good |
| Yang, 2011 | SEER, 1988-2007 | NR | 364 (0.5) | 76106 (98.6) | NA | NR | NR | Good |
| Zell, 2008 | CCR, 1993-2003 | NR | 127 (0.3) | 36694 (94.0) | NA | NR | NR | Fair |
| *Melanoma-specific survival* | | | | | | | | |
| Abdel-Rahman, 2019 | SEER, 2000-2005 | NR | 1079 (0.4) | 240818 (95.7) | NA | NR | NR | Fair |
| Allan, 2015 | SEER, 1974-2009 | ≤19 | 3 (0.4) | 670 (92.5) | 5-year | 100 | 97.4 | Fair |
| Asgari, 2017 | KPNC, 2004-2012 | NR | 5 (4.1) | 92 (74.8) | NA | NR | NR | Good |
| Baldwin, 2016 | OCCR, 2000-2008 | NR | 36 (0.5) | 6480 (94.3) | NR | 80.6 | 83.0 | Good |
| Bradford, 2009 | SEER, 1986-2005 | NR | 108 (7.6) | 1000 (70.8) | 5-year | 77.2 | 82.6 | Fair |
|  |  |  |  |  | 10-year | 71.5 | 69.4 |  |
| Collins, 2011 | SEER, 1973-2004 | NR | 789 (0.5) | 148833 (98.5) | 5-year | 69.8 | 68.4 | Good |
|  |  |  |  |  | 10-year | 69.8 | 66.5 |  |
| Cormier, 2006 | SEER, 1992-2002 | NR | 251 (0.5) | 48143 (96.7) | NA | NR | NR | Fair |
| De Lacerda, 2019 | SEER, 2000-2013 | ≥20 | 867 (0.5) | 167604 (95.4) | 5-year | 66.5 | 87.4 | Poor |
| Du, 2011 | SEER, 1973-2003 | ≥25 | 922 (7.0) | 10749 (81.2) | NA | NR | NR | Good |
| Hu, 2013 | SEER, 1988-1999 | NR | 176 (0.6) | 27940 (95.1) | 5-year | 70.8 | 87.8 | Fair |
| Jemal, 2004 | SEER/NPCR, 1999-2013 | NR | NR | NR | 5-year | 77.7 (males), 71.5 (females) | 86.5 (males), 92.2 (females) | Fair |
| Jemal, 2017 | SEER/NPCR, 1975-2001 | NR | NR | NR | 5-year | 67.1 | 89.6 | Fair |
| Kachare, 2014 | SEER, 2003-2008 | ≥18 | 43 (0.5) | 7692 (97.2) | 5-year | 78.7 | 84.8 | Good |
| Lam, 2018 | SEER, 2004-2008 | ≤21 | 6 (0.5) | 1067 (85.0) | NA | NR | NR | Fair |
| Mahendraraj, 2017 | SEER, 1988-2011 | NR | 1106 (0.5) | 212721 (99.5) | 5-year | 70.0 | 89.0 | Poor |
| Pollack, 2011 | SEER, 1992-2005 | ≥15 | 318 (0.5) | 65174 (95.2) | 5-year | 72.2 | 89.2 | Fair |
|  |  |  |  |  | 10-year | 68.2 | 85.0 |  |
| Reyes-Ortiz, 2006 | SEER, 1988-1999 | ≥65 | 148 (0.6) | 22227 (96.4) | NA | NR | NR | Poor |
| Shaikh, 2016 | SEER, 1989-2009 | NR | 445 (0.5) | 95091 (96.5) | NA | NR | NR | Fair |
| Stokes, 2014 | SEER, 2004-2009 | NR | 27 (0.2) | 11637 (95.4) | NA | NR | NR | Good |
| Ward-Peterson, 2016 | SEER, 1982-2011 | ≥18 | 3070 (1.7) | 169844 (94.4) | NA | NR | NR | Good |
| Wu, 2011 | SEER/NPCR, 1999-2005 | NR | 1396 (0.5) | 274052 (97.0) | 5-year | 78.2 | 90.0 | Fair |
| Zell, 2008 | CCR, 1993-2003 | NR | 127 (0.3) | 36694 (94.0) | NA | NR | NR | Fair |

**Table S4**: Studies reporting % survival for Hispanic patients compared to non-Hispanic white patients

| **Study** | **n** | **Duration of follow-up** | **% survival in Hispanic patients** | **% survival in NWH patients** |
| --- | --- | --- | --- | --- |
| *Overall survival* | | | | |
| DeRouen, 2017 | 27111 | 10 years | 99.7 | 98.8 |
| *Melanoma-specific survival* | | | | |
| Allan, 2015 | 35 | 5 years | 100 | 97.4 |
| Baldwin, 2016 | 62 | NR | 87.1 | 83.0 |
| Bradford, 2009 | 151 | 5 years | 72.8 | 82.6 |
|  |  | 10 years | 57.3 | 69.4 |
| Cormier, 2006 | 932 | NR | 86.1 | 91.5 |
| De Lacerda, 2019 | 5743 | 5 years | 78.8 | 87.4 |
| Jemal, 2004 | NR | 5 years | 77.1 | 86.5 |
| Jemal, 2017 | NR | 5 years | 84.6 | 89.6 |
| Pollack, 2011 | 2104 | 5 years | 83.3 | 89.2 |
| Wu, 2011 | 5613 | 10 years | 79.5 | 85.0 |
| Zell, 2008 | 1966 | 5 years | 87.0 | 90.0 |

**Table S5**: Studies reporting % survival for Asian and Pacific Islander patients compared to non-Hispanic white patients

| **Study** | **n** | **Duration of follow-up** | **% survival in A/PI patients** | **% survival in NWH patients** |
| --- | --- | --- | --- | --- |
| *Overall survival* | | | | |
| DeRouen, 2017 | 9571 | 10 years | 99.9 | 98.8 |
| *Melanoma-specific survival* | | | | |
| Allan, 2015 | 2 | 5 years | 100 | 97.4 |
| Baldwin, 2016 | 17 | NR | 100 | 83.0 |
| Bradford, 2009 | 70 | 5 years | 70.2 | 82.6 |
|  |  | 10 years | 54.1 | 69.4 |
| Cormier, 2006 | 394 | NR | 85.3 | 91.5 |
| De Lacerda, 2019 | 1130 | 5 years | 73.5 | 87.4 |
| Jemal, 2004 | NR | 5 years | 80.1 | 86.5 |
| Jemal, 2017 | NR | 5 years | 84.8 | 89.6 |
| Pollack, 2011 | 593 | 5 years | 79.2 | 89.2 |
|  |  | 10 years | 71.7 | 85.0 |
| Shin, 2009 | 1237 | 5 years | 70.7 | 75.2 |
| Wu, 2011 | 947 | 5 years | 80.7 | 90.0 |

**Table S6**: Studies reporting % survival for American Indian and Alaskan Native patients compared to non-Hispanic white patients

| **Study** | **n** | **Duration of follow-up** | **% survival in AI/AN patients** | **% survival in NWH patients** |
| --- | --- | --- | --- | --- |
| *Overall survival* | | | | |
| - | - | - | - | - |
| *Melanoma-specific survival* | | | | |
| Baldwin, 2016 | 340 | NR | 89.7 | 83.0 |
| Cormier, 2006 | 52 | NR | 82.7 | 91.5 |
| De Lacerda, 2019 | 359 | 5 years | 81.1 | 87.4 |
| Jemal, 2004 | NR | 5 years | 80.1 | 86.5 |
| Jemal, 2017 | NR | 5 years | 84.8 | 89.6 |
| Pollack, 2011 | 124 | 5 years | 79.5 | 89.2 |
|  |  | 10 years | 74.6 | 85.0 |
| Wu, 2011 | 567 | 5 years | 84.9 | 90.0 |

**Table S7**: PRISMA checklist

| **Section/topic** | **#** | **Checklist item** | **Reported on page #** |
| --- | --- | --- | --- |
| **TITLE** | | |  |
| Title | 1 | Identify the report as a systematic review, meta-analysis, or both. | 1 |
| **ABSTRACT** | | |  |
| Structured summary | 2 | Provide a structured summary including, as applicable: background; objectives; data sources; study eligibility criteria, participants, and interventions; study appraisal and synthesis methods; results; limitations; conclusions and implications of key findings; systematic review registration number. | 2 |
| **INTRODUCTION** | | |  |
| Rationale | 3 | Describe the rationale for the review in the context of what is already known. | 4 |
| Objectives | 4 | Provide an explicit statement of questions being addressed with reference to participants, interventions, comparisons, outcomes, and study design (PICOS). | 4 |
| **METHODS** | | |  |
| Protocol and registration | 5 | Indicate if a review protocol exists, if and where it can be accessed (e.g., Web address), and, if available, provide registration information including registration number. | 1, 4 |
| Eligibility criteria | 6 | Specify study characteristics (e.g., PICOS, length of follow-up) and report characteristics (e.g., years considered, language, publication status) used as criteria for eligibility, giving rationale. | 4, 5 |
| Information sources | 7 | Describe all information sources (e.g., databases with dates of coverage, contact with study authors to identify additional studies) in the search and date last searched. | 4, 5 |
| Search | 8 | Present full electronic search strategy for at least one database, including any limits used, such that it could be repeated. | 4, Table S1-S2 |
| Study selection | 9 | State the process for selecting studies (i.e., screening, eligibility, included in systematic review, and, if applicable, included in the meta-analysis). | 5 |
| Data collection process | 10 | Describe method of data extraction from reports (e.g., piloted forms, independently, in duplicate) and any processes for obtaining and confirming data from investigators. | 5 |
| Data items | 11 | List and define all variables for which data were sought (e.g., PICOS, funding sources) and any assumptions and simplifications made. | 5 |
| Risk of bias in individual studies | 12 | Describe methods used for assessing risk of bias of individual studies (including specification of whether this was done at the study or outcome level), and how this information is to be used in any data synthesis. | 5 |
| Summary measures | 13 | State the principal summary measures (e.g., risk ratio, difference in means). | 6 |
| Synthesis of results | 14 | Describe the methods of handling data and combining results of studies, if done, including measures of consistency (e.g., I^2^) for each meta-analysis. | 6 |

| **Section/topic** | **#** | **Checklist item** | **Reported on page #** |
| --- | --- | --- | --- |
| Risk of bias across studies | 15 | Specify any assessment of risk of bias that may affect the cumulative evidence (e.g., publication bias, selective reporting within studies). | N/A |
| Additional analyses | 16 | Describe methods of additional analyses (e.g., sensitivity or subgroup analyses, meta-regression), if done, indicating which were pre-specified. | N/A |
| **RESULTS** | | |  |
| Study selection | 17 | Give numbers of studies screened, assessed for eligibility, and included in the review, with reasons for exclusions at each stage, ideally with a flow diagram. | 6, Fig. 1 |
| Study characteristics | 18 | For each study, present characteristics for which data were extracted (e.g., study size, PICOS, follow-up period) and provide the citations. | 6, Table 1 |
| Risk of bias within studies | 19 | Present data on risk of bias of each study and, if available, any outcome level assessment (see item 12). | Table 1 |
| Results of individual studies | 20 | For all outcomes considered (benefits or harms), present, for each study: (a) simple summary data for each intervention group (b) effect estimates and confidence intervals, ideally with a forest plot. | 7-8 |
| Synthesis of results | 21 | Present results of each meta-analysis done, including confidence intervals and measures of consistency. | 7-9, Fig. 2-3 |
| Risk of bias across studies | 22 | Present results of any assessment of risk of bias across studies (see Item 15). | N/A |
| Additional analysis | 23 | Give results of additional analyses, if done (e.g., sensitivity or subgroup analyses, meta-regression [see Item 16]). | N/A |
| **DISCUSSION** | | |  |
| Summary of evidence | 24 | Summarize the main findings including the strength of evidence for each main outcome; consider their relevance to key groups (e.g., healthcare providers, users, and policy makers). | 9-11 |
| Limitations | 25 | Discuss limitations at study and outcome level (e.g., risk of bias), and at review-level (e.g., incomplete retrieval of identified research, reporting bias). | 10 |
| Conclusions | 26 | Provide a general interpretation of the results in the context of other evidence, and implications for future research. | 10-11 |
| **FUNDING** | | |  |
| Funding | 27 | Describe sources of funding for the systematic review and other support (e.g., supply of data); role of funders for the systematic review. | 1 |

*From:*  Moher D, Liberati A, Tetzlaff J, Altman DG, The PRISMA Group (2009). Preferred Reporting Items for Systematic Reviews and Meta-Analyses: The PRISMA Statement. PLoS Med 6(7): e1000097. doi:10.1371/journal.pmed1000097
